# Exploration of predictors of benefit from nivolumab monotherapy for patients with pretreated advanced gastric and gastroesophageal junction cancer: post hoc subanalysis from the ATTRACTION-2 study

**DOI:** 10.1007/s10120-021-01230-4

**Published:** 2021-09-04

**Authors:** Yoon-Koo Kang, Satoshi Morita, Taroh Satoh, Min-Hee Ryu, Yee Chao, Ken Kato, Hyun Cheol Chung, Jen-Shi Chen, Kei Muro, Won Ki Kang, Kun-Huei Yeh, Takaki Yoshikawa, Sang Cheul Oh, Li-Yuan Bai, Takao Tamura, Keun-Wook Lee, Yasuo Hamamoto, Jong Gwang Kim, Keisho Chin, Do-Youn Oh, Keiko Minashi, Jae Yong Cho, Masahiro Tsuda, Hiroki Sameshima, Li-Tzong Chen, Narikazu Boku

**Affiliations:** 1 Department of Oncology, Asan Medical Center, University of Ulsan College of Medicine, Seoul, South Korea; 2 Department of Biomedical Statistics and Bioinformatics, Kyoto University Graduate School of Medicine, Kyoto, Japan; 3 Frontier Science for Cancer and Chemotherapy, Osaka University Graduate School of Medicine, Suita, Japan; 4 Department of Oncology, Taipei Veterans General Hospital, Taipei, Taiwan; 5 Division of Gastrointestinal Medical Oncology, National Cancer Center Hospital, Tokyo, Japan; 6 Department of Internal Medicine, Yonsei Cancer Center, Yonsei University College of Medicine, Seoul, South Korea; 7 Division of Hematology and Oncology, Department of Internal Medicine, Linkou Chang Gung Memorial Hospital, Chang Gung University, Taoyuan, Taiwan; 8 Department of Clinical Oncology, Aichi Cancer Center Hospital, Nagoya, Japan; 9 Division of Hematology-Oncology, Department of Medicine, Samsung Medical Center, Sungkyunkwan University School of Medicine, Seoul, South Korea; 10 Department of Oncology, National Taiwan University Hospital, Taipei, Taiwan; 11 Department of Gastrointestinal Surgery, Kanagawa Cancer Center, Yokohama, Japan; 12 Division of Hematology and Oncology, Department of Internal Medicine, College of Medicine, Korea University, Seoul, South Korea; 13 Division of Hematology and Oncology, Department of Internal Medicine, China Medical University Hospital, China Medical University, Taichung, Taiwan; 14 Department of Medical Oncology, Faculty of Medicine, Kindai University, Osakasayama, Japan; 15 Division of Hematology and Oncology, Department of Internal Medicine, Seoul National University Bundang Hospital, Seoul National University College of Medicine, Seongnam, South Korea; 16 Keio Cancer Center, Keio University School of Medicine, Tokyo, Japan; 17 Kyungpook National University School of Medicine, Daegu, South Korea; 18 Department of Gastroenterology, Cancer Institute Hospital of the Japanese Foundation for Cancer Research, Tokyo, Japan; 19 Seoul National University Hospital, Cancer Research Institute, Seoul National University College of Medicine, Integrated Major in Innovative Medical Science, Seoul National University Graduate School, Seoul, South Korea; 20 Department of Clinical Trial Promotion, Chiba Cancer Center, Chiba, Japan; 21 Department of Medical Oncology, Gangnam Severance Hospital, Yonsei University College of Medicine, Seoul, South Korea; 22 Department of Gastroenterological Oncology, Hyogo Cancer Center, Akashi, Japan; 23 Medical Affairs, Ono Pharmaceutical, Co. Ltd., Osaka, Japan; 24 National Institute of Cancer Research, National Health Research Institutes, Tainan, Taiwan; 25 Kaohsiung Medical University Hospital, Kaohsiung Medical University, Kaohsiung, Taiwan; 26 Present Address: Department of Gastric Surgery, National Cancer Center Hospital, Tokyo, Japan; 27 Present Address: Department of Medical Oncology, Kindai University Nara Hospital, Ikoma, Japan

**Keywords:** Gastric cancer, Gastroesophageal junction, Nivolumab, Early progression, Benefit predictors

## Abstract

**Background:**

The phase 3 ATTRACTION-2 study demonstrated that nivolumab monotherapy was superior to placebo for patients with pretreated advanced gastric or gastroesophageal junction cancer, but early progression of tumors in some patients was of concern.

**Methods:**

This post hoc analysis statistically explored the baseline characteristics of the ATTRACTION-2 patients and extracted a single-factor and double-factor combinations associated with early disease progression or early death. In the extracted patient subgroups, the 3-year restricted mean survival times of progression-free survival and overall survival were compared between the nivolumab and placebo arms.

**Results:**

Two single factors (age and peritoneal metastasis) were extracted as independent predictors of early progression, but none of them, as a single factor, stratified patients into two subgroups with significant differences in restricted mean survival time. In contrast, two double-factor combinations (serum sodium level and white blood cell count; serum sodium level and neutrophil–lymphocyte ratio) stratifying patients into two subgroups with significant differences in the restricted mean survival time were extracted. Additional exploratory analysis of a triple-factor combination showed that patients aged < 60 years with peritoneal metastasis and low serum sodium levels (approximately 7% of all patients) might receive less benefit from nivolumab, and patients aged ≥ 60 years with no peritoneal metastasis and normal serum sodium levels might receive higher benefit.

**Conclusions:**

A combination of age, peritoneal metastasis, and serum sodium level might predict benefit from nivolumab as salvage therapy in advanced gastric or gastroesophageal junction cancer patients, especially less benefit for patients having all three risk factors.

**Supplementary Information:**

The online version contains supplementary material available at 10.1007/s10120-021-01230-4.

## Background

Immune checkpoint inhibitors (ICIs) have led to substantial breakthroughs in cancer therapy [1]. By blocking an immune checkpoint pathway, ICIs promote the activation of cytotoxic T lymphocytes, thereby facilitating the removal of tumor cells. ICIs have been approved for therapy of multiple cancers. A remarkable advantage of ICI therapy is its long-term survival benefit. However, it has become evident that some patients respond poorly to ICI therapy, and immune-related adverse events (irAEs) are of great concern, particularly for non-responders [2–4]. Treatment effect predictors are useful in the decision-making in daily practice of clinicians for optimizing patient benefit [5–7].

Monoclonal antibodies against human programmed-death receptor 1 (PD-1), such as nivolumab and pembrolizumab, inhibit a PD-1-mediated suppression signal of cytotoxic T lymphocytes [8]. Nivolumab and pembrolizumab have been proven to be effective for several types of cancer, including malignant melanoma, non-small cell lung carcinoma, and gastric cancer. The most frequently evaluated effect predictor for these PD-1 blockades is the programmed-death ligand 1 (PD-L1) expression level because PD-L1 binds to PD-1 on T lymphocytes to induce the PD-1-mediated suppression signal [6, 9]. Indeed, several approved indications are linked to testing PD-L1 expression on tumor cells and tumor-infiltrating immune cells [7]. On the other hand, some clinical trials have reported a lower correlation between the efficacy of PD-1 blockades and PD-L1 expression. Thus, it remains a controversial issue whether PD-L1 expression is a widely applicable predictor of benefit from PD-1 blockades [7]. Likewise, other potential biomarkers, such as the tumor mutation burden, microsatellite instability, and CD8^+^ T lymphocytes, have been evaluated, and some of them are linked to approved indications [10, 11], but no powerful predictors of PD-1 blockade efficacy have been discovered [12].

The phase 3 ATTRACTION-2 study evaluated the efficacy and safety of nivolumab monotherapy as third-line or later-line treatment in advanced gastric or gastroesophageal junction (G/GEJ) cancer patients [13]. In ATTRACTION-2, nivolumab significantly reduced risks of death and disease progression compared with placebo [13]. A subsequent follow-up analysis showed a better 3-year overall survival (OS) rate in the nivolumab arm [14]. Although nivolumab apparently provides benefit to G/GEJ cancer patients, early disease progression within two months was observed in approximately half of both the nivolumab- and placebo-treated patients, suggesting that some patients benefited poorly from nivolumab. In ATTRACTION-2, PD-L1 expression by tumor cells was not predictive of treatment efficacy, and other potential biomarkers could not be evaluated due to data limitations [13].

To help clinician’s decision-making, this post hoc subanalysis in ATTRACTION-2 aimed to explore the baseline characteristics of patients that were associated with low and high benefit from nivolumab as salvage therapy for gastric cancer. Conventional univariable and multivariable analyses have been often used to identify predictive biomarkers. However, since these conventional analyses consider an outcome of only a single treatment arm, prognostic but not predictive factors may be extracted. Furthermore, combinations of two or more factors are rarely analyzed. Recently, several statistical methods have been developed to overcome these limitations [15], one of which is the “BaPoFi” method [16]. To explore subgroups that are most likely to benefit from one of two treatment arms in a randomized trial, the BaPoFi method can evaluate the difference in treatment effects between two treatment arms in all possible subgroups classified by a combination of multiple factors as well as by a single factor. In this study, BaPoFi analysis, as well as conventional univariable and multivariable analyses, was performed to extract predictive factors.

The hazard ratio is a well-established evaluation statistic, but the underlying proportional hazards assumption may be violated in ICI therapy [17]. Therefore, the restricted mean survival time (RMST) has been proposed as an alternative and statistically valid method for assessing the effectiveness of ICI therapy [18, 19]. To evaluate the treatment effects of subgroups in this post hoc subanalysis, we compared the estimated RMST of the progression-free survival (PFS) and that of the OS between the nivolumab and placebo arms.

## Methods

### Study design

ATTRACTION-2 was a randomized, double-blind, placebo-controlled, multicenter phase 3 clinical trial conducted in Japan, South Korea, and Taiwan, and was registered with ClinicalTrials.gov, number NCT02267343. The detailed design of ATTRACTION-2 was described previously [13]. Additional informed consent specific for this post hoc study was not obtained from the ATTRACTION-2 participants.

### Patients

A total of 493 metastatic G/GEJ cancer patients were enrolled in ATTRACTION-2, 330 and 163 of whom were randomly allocated to receive nivolumab and placebo, respectively. Two patients in the placebo arm were excluded from this post hoc subanalysis due to the study drugs not being administered to them. Because patient data, such as body mass index and C-reactive protein levels, were missing for some patients, the BaPoFi and RMST analyses included 318 and 155 patients in the nivolumab and placebo arms, respectively, all of whom have all required data (Online Resource Fig. S1a).

### Variables

In this post hoc subanalysis, we included patients with OS and PFS data of a minimum 3-year follow-up [14]. Baseline characteristics, clinical history, and clinical laboratory values of the patients were used in the statistical analyses (Online Resource Table S1). These data were recorded at enrollment to ATTRACTION-2, except for primary tumor site and histological classification that were obtained at diagnosis of G/GEJ cancer. The original data for metastasis were those collected at diagnosis of G/GEJ cancer, and therefore, we generated data on metastasis using computed tomography or magnetic resonance imaging records regarding target and non-target legions at enrollment to the study. The cutoff value for each laboratory factor was institutional lower or upper limits, medians, tertiles, or clinically meaningful values (Online Resource Table S1).

### Statistical methods for extraction

To extract independent factors associated with disease progression, we used two models for conventional univariable and multivariable analyses. Logistic regression analyses assessed PFS within 8 weeks in the nivolumab arm, when the Kaplan–Meier curves of PFS began to markedly separate between the nivolumab and placebo arms. We also assessed PFS over time by Cox regression analyses. The multivariable analysis assessed factors that was identified with *p* values less than 0.05 in the preceding univariable analysis. Then, we focused on the independent factors related with these two outcomes of PFS that were extracted from both multivariable logistic and Cox analyses with *p* values less than 0.05. Here we used PFS data but not OS data because we focused on early progression rather than OS.

To consider double-factor combinations, we conducted the BaPoFi method. The BaPoFi method considers the problem to identify effect predictors using Bayesian additive regression trees (BART) and evaluate subgroups with differential effects as a Bayesian decision problem. In the BART computation, we assume a conjugate normal prior for the mean parameters of the terminal nodes and assume a conjugate inverse-chi-squared prior for the variance parameter. In this study, the BaPoFi analysis considered the PFS rates at 8 weeks and OS rates at 5.26 months between the nivolumab and placebo arms as independent binary definitions: survival and non-survival. The OS rates of 5.26 months was the median OS in the nivolumab arm of the total patient population. Expected utility with respect to future outcomes were evaluated for each single factor and for a combination of double factors. Single factors and combinations of double factors ranked at the top five of preferable outcomes were extracted. Of note, the BaPoFi method focuses on a meaningful difference between the two treatment arms, thereby potentially avoiding extraction of mere prognostic factors even if OS rates are used.

### Statistical methods for validation

A total of 318 patients in the nivolumab arm and 155 patients in the placebo arm were classified into two categories based on the cutoff value of a single factor: a low-benefit group and high-benefit group (Online Resources Fig. S1a and b). A double-factor combination classified patients into three categories: a low-benefit group that included patients with two risk factors in the double-factor combination, a high-benefit group that included patients with no risk factors in the double-factor combination, and the others with either one risk factor (Online Resources Fig. S1a and b). To assess the benefit from nivolumab in each subgroup, Kaplan–Meier curves of OS and PFS were estimated between the nivolumab and placebo arms, followed by estimation of the RMST at 36 months after the first dose (Online Resources Fig. S1c). Here, we considered a difference of RMST, but not a ratio of RMST, as the effect measure because a treatment benefit may be intuitively understandable for clinicians and patients. We defined ∆RMST as the difference in the estimated RMST between the nivolumab and placebo arms (nivolumab minus placebo), which could be considered the potential benefit from nivolumab (Online Resources Fig. S1c). Also, we defined ∆∆RMST as the difference in the ∆RMST between the low- and high-benefit groups (high minus low), which would reflect the difference in benefit from nivolumab between them (Online Resources Fig. S1d). Thus, factors associated with a larger ∆∆RMST of both PFS and OS are better predictors of benefit from nivolumab. A *p* value below 0.05 was considered to indicate statistical significance. Best overall responses and objective response rates in certain patient subgroups were calculated as described previously [13].

## Results

### RMST analysis for the entire ATTRACTION-2 patient population

Estimated RMSTs at 36 months of OS and PFS for the entire intention-to-treat population in the nivolumab arm (*N* = 330) were 9.6 and 4.7 months, respectively, and those in the placebo arm (*N* = 163) were 6.0 and 2.4 months, respectively. The ∆RMSTs of OS and PFS between the nivolumab and placebo arms were 3.6 months (95% confidence interval [CI], 2.10–5.05; *p* < 0.0001) and 2.3 months (95% CI, 1.37–3.22; *p* < 0.0001), respectively, indicating that the RMST analysis demonstrated significant improvement by nivolumab in both OS and PFS in the entire ATTRACTION-2 patient population.

### Conventional univariable and multivariable analyses

Conventional univariable logistic and Cox regression analyses individually identified around 20 factors associated with disease progression in the nivolumab arm (Online Resource Tables S2 and S3). Because multiple cutoff values were extracted for age (50, 60, 65, and 70 years), we conducted Kaplan–Meier and RMST analyses of PFS for the patient subgroups classified according to these cutoff values. As a result, an age of 60 years, but not ages of 50, 65, or 70 years, classified patients into low- and high-benefit groups showing statistically significant differences in ∆∆RMST (Online Resource Table S4). Thus, conventional multivariable analyses were conducted using 60 years as the cutoff value for age. The multivariable logistic regression analysis identified age, peritoneal metastasis, and liver metastasis as risk factors of disease progression within 8 weeks and the multivariable Cox regression analysis identified age, lymph node metastasis, peritoneal metastasis, white blood cell count, and lactate dehydrogenase value as being independent factors associated with disease progression (Online Resource Tables S2 and S3). Age and peritoneal metastasis that were extracted from both multivariable analyses were used for subsequent analyses (Table 1).

**Table 1:** Independent factors identified in both conventional logistic and Cox regression multivariable analyses as being significantly associated with early progression in the nivolumab arm

Factors [Cutoff]	∆∆RMST^a^ of OS (95% CI), months	∆∆RMST^a^ of PFS (95% CI), months
Age [< 60 vs. ≥ 60]	2.7 (− 0.09–5.47) *p* = 0.0584	**1.9 (0.14–3.59)** ***p* = 0.0341**
Metastasis [Peritoneal metastasis vs. No]	**4.2 (1.20–7.30)** ***p* = 0.0063**	0.9 (− 0.90–2.76) *p* = 0.3202

Conventional multivariable analysis was conducted, which included the factors identified in the preceding univariable analysis as being significantly associated with early progression in the nivolumab arm

*CI* confidence interval, *OS* overall survival, *PFS* progression-free survival, *RMST* restricted mean survival time

^a^Statistically significant differences (*p* < 0.05) are highlighted in bold. The definition of ∆∆RMST is described in “Methods”

The Kaplan–Meier and RMST analyses of OS and PFS for the patient subgroups classified by these independent factors indicated that the ∆∆RMST of PFS between the low-benefit (< 60 years) and high-benefit (≥ 60 years) groups classified by age was 1.9 months and was significantly different, whereas ∆∆RMST of OS did not show a statistically significant difference between the two groups (Table 1; Online Resource Fig. S2). A significant difference in the ∆∆RMST between the low-benefit (with peritoneal metastasis) and high-benefit (without peritoneal metastasis) groups classified by peritoneal metastasis was observed for OS (4.2 months) but not for PFS. Thus, as a single factor, none of these independent factors showed statistically significant differences between the two groups in both ∆∆RMST values of OS and PFS.

### BaPoFi analysis

**Fig. 1:**
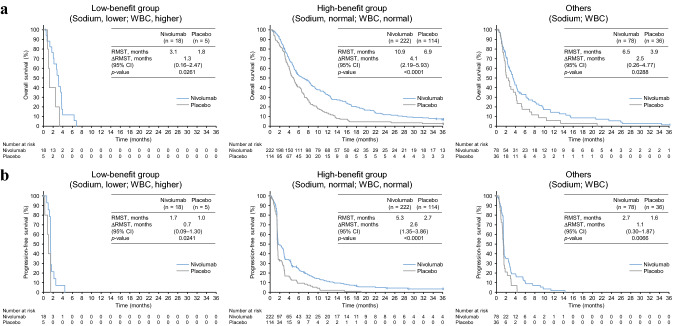
Classification by the double-factor combination of serum sodium level and WBC. The Kaplan–Meier curves of the OS (**a**) and PFS (**b**) with the RMST values in the low-benefit group (left), in the high-benefit group (center), and in the others (right) are shown. *CI* confidence interval, *OS* overall survival, *PFS* progression-free survival, *RMST* restricted mean survival time, *∆RMST* difference in RMST between the nivolumab and placebo arms, *WBC* white blood cell count

**Fig. 2:**
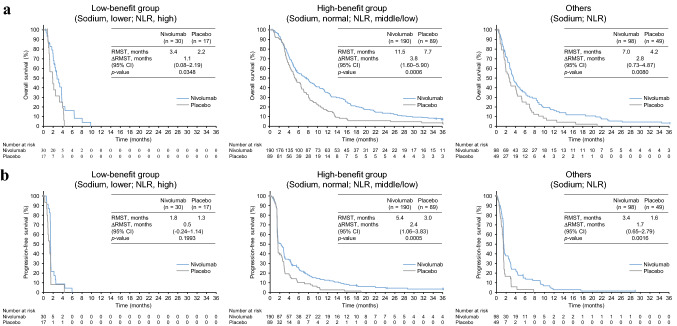
Classification by the double-factor combination of serum sodium level and NLR. The Kaplan–Meier curves with the RMST are shown as described in Fig. 1. *NLR* neutrophil–lymphocyte ratio

Besides the conventional multivariable analyses, we conducted an analysis using the BaPoFi method to identify double-factor combinations associated with preferable OS or PFS in the nivolumab arm compared with the placebo arm because the BaPoFi method has an advantage on exploring high-benefit groups rather than low-benefit groups. Four double-factor combinations were extracted in both OS and PFS analyses (Online Resource Table S5 shows the top five subgroups). We classified patients into three subgroups using the identified double-factor combinations: a high-benefit group with the two preferable factors (for example, normal serum sodium level and normal white blood cell count [WBC]), a low-benefit group with two risk factors (for example, lower serum sodium level and higher WBC), and others with one preferable and one risk factors (Online Resource Fig. S1b). The RMST analyses showed statistically significant differences in the ∆∆RMST of both OS and PFS between the low- and high-benefit groups in two of the double-factor combinations: The ∆∆RMST values of OS and PFS between low- and high-benefit groups classified by the combination of serum sodium level (lower vs. normal range) and WBC (higher vs. normal range) were 2.7 and 1.9 months, respectively, and those classified by the combination of serum sodium level (lower vs. normal range) and neutrophil–lymphocyte ratio ([NLR]; highest tertile vs. middle or lowest tertile) were 2.6 and 2.0 months, respectively (Table 2; Figs. 1 and 2; Online Resource Fig. S3).

**Table 2:** Top four double-factor combinations identified in both the OS and PFS subanalyses in the BaPoFi analysis

Factors [classification], low- vs. high-benefit groups	*n* (nivolumab/placebo)	∆∆RMST^a^ of OS (95% CI), months	∆∆RMST^a^ of PFS (95% CI), months
Na [Lower] + WBC [Higher] vs Na [Normal] + WBC [Normal]	18/5 vs 222/114	**2.7 (0.55–4.95)** ***p* = 0.0143**	**1.9 (0.52–3.31)** ***p* = 0.007**
Na [Lower] + NLR [High] vs Na [Normal] + NLR [Middle/low]	30/17 vs 190/89	**2.6 (0.23–5.01)** ***p* = 0.0319**	**2.0 (0.44–3.54)** ***p* = 0.0116**
Na [Lower] + CL [Lower] vs Na [Normal] + CL [Normal]	29/12 vs 246/123	1.3 (− 1.82–4.49) *p* = 0.4077	1.1 (− 0.62–2.75) *p* = 0.2141
CL [Lower] + NLR [High] vs CL [Normal] + NLR [Middle/low]	26/16 vs 199/96	1.8 (− 1.02–4.65) *p* = 0.2102	1.6 (− 0.03–3.30) *p* = 0.055

*CI* confidence interval, *CL* serum chloride level, *Na* serum sodium level, *NLR* neutrophil–lymphocyte ratio, *OS* overall survival, *PFS* progression-free survival, *RMST* restricted mean survival time, *WBC* white blood cell count

^a^Statistically significant differences (*p* < 0.05) are highlighted in bold. The definition of ∆∆RMST is described in “Methods”

### Exploratory analyses

The BaPoFi analysis-extracted combinations included four single factors (serum sodium level, serum chloride level, WBC, and NLR; all were laboratory data), which were totally different from the two independent factors extracted in the conventional multivariable analyses (age and peritoneal metastasis; these were patient’s condition). However, the ∆∆RMST values of OS and PFS in patient subgroups classified by the BaPoFi analysis-extracted combinations were numerically smaller than those in the subgroup classified by the combination of age and peritoneal metastasis. To test the possibility that any double-factor combinations of these six extracted factors, 15 combinations in total, defined the low- and high-benefit groups with a larger ∆∆RMST, we conducted a further exploratory analysis for additional 11 possible double-factor combinations; the 4 combinations identified by the BaPoFi analysis were already analyzed above. Among them, seven combinations of double factors showed statistically significant differences in the ∆∆RMST values of both OS and PFS between the low- and high-benefit groups (Tables 2, 3). The largest ∆∆RMST of OS was 6.8 months, which was shown for the patient subgroups classified using an age of 60 years and serum sodium level. In addition, while the ∆∆RMST values of OS in the top three combinations (age and serum sodium level, age and peritoneal metastasis, and peritoneal metastasis and serum sodium level) were remarkably larger than those in other combinations, these constituents mutually overlapped.

**Table 3:** Double-factor combinations showing statistically significant differences in ∆∆RMST of both OS and PFS between the low- and high-benefit groups in the exploratory analysis

Factors [classification], low- vs. high-benefit groups	*n* (nivolumab/placebo)	RMST^a^ of OS (95% CI), months	∆∆RMST^a^ of PFS (95% CI), months
Age [< 60] + Na [Lower] vs Age [≥ 60] + Na [Normal]	27/13 vs 154/77	**6.8 (2.57–10.97)** ***p* = 0.0016**	**2.8 (0.98–4.55)** ***p* = 0.0024**
Age [< 60] + P-meta [Yes] vs Age [≥ 60] + P-meta [No]	77/43 vs 93/40	**6.5 (2.43–10.52)** ***p* = 0.0017**	**2.8 (0.58–5.08)** ***p* = 0.0138**
P-meta [Yes] + Na [Lower] vs P-meta [No] + Na [Normal]	37/21 vs 130/57	**6.1 (2.52–9.74)** ***p* = 0.0009**	**2.1 (0.19–3.95)** ***p* = 0.0312**
Age [< 60] + CL [Lower] vs Age [≥ 60] + CL [Normal]	22/8 vs 162/80	**4.4 (1.46–7.41)** ***p* = 0.0034**	**2.3 (0.61–4.01)** ***p* = 0.0077**
CL [Lower] + WBC [Higher] vs CL [Normal] + WBC [Normal]	12/7 vs 229/124	**3.5 (1.51–5.58)** ***p* = 0.0006**	**2.4 (1.17–3.71)** ***p* = 0.0002**

*CI* confidence interval, *CL* serum chloride level, *Na* serum sodium level, *NLR* neutrophil–lymphocyte ratio, *OS* overall survival, *P-meta* peritoneal metastasis, *PFS* progression-free survival, *RMST* restricted mean survival time, *WBC* white blood cell count

^a^Statistically significant differences (*p* < 0.05) are highlighted in bold. The definition of ∆∆RMST was described in “Methods”

**Fig. 3:**
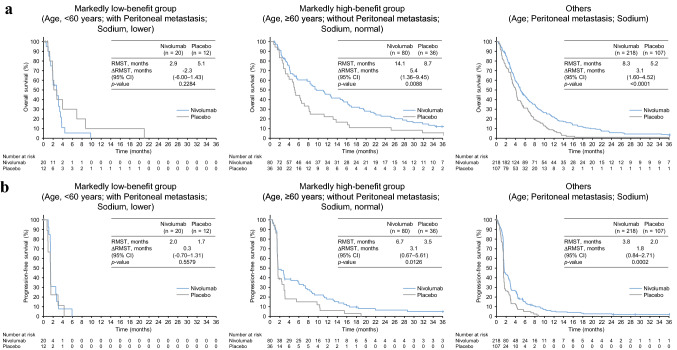
Classification by the triple-factor combination of age, peritoneal metastasis, and serum sodium level. The Kaplan–Meier curves with the RMST are shown as described in Fig. 1

Thus, in a further exploratory analysis, we evaluated a combination of the top three factors: age, peritoneal metastasis, and serum sodium level. Patients younger than 60 years with peritoneal metastasis and with a lower serum sodium level who were classified into the markedly low-benefit group accounted for 6.3% and 7.7% of all patients in the nivolumab and placebo arms, respectively, and patients aged 60 years or older without peritoneum metastasis and with normal serum sodium level who were classified into the markedly high-benefit group accounted for 25.2% and 23.2% of all patients in the nivolumab and placebo arms, respectively. The ∆∆RMST values of OS and PFS between the markedly low- and high-benefit groups in these triple-factor-classified subgroups were 7.7 and 2.8 months, respectively, which showed the largest difference with statistical significance obtained in this study (Table 4; Fig. 3). No responders were included in the markedly low-benefit group and the objective response rate in the nivolumab arm of the markedly high-benefit group was 21% (Table 4).

**Table 4:** Comparison between the markedly low- and high-benefit groups classified by the triple-factor combination of age, peritoneal metastasis, and serum sodium level

Factors [classification]	Markedly low-benefit group		Markedly high-benefit group	
	Age [< 60] + P-meta [Yes] + Na [Lower]		Age [≥ 60] + P-meta [No] + Na [Normal]	
Treatment arm	Nivolumab	Placebo	Nivolumab	Placebo
*n*	20	12	80	36
∆∆RMST^a^ of OS—months, (95% CI)	**7.7** **(2.20–13.18);** ***p*** **=** **0.0061**			
∆∆RMST^a^ of PFS—months, (95% CI)	**2.8** **(0.18–5.50);** ***p*** **=** **0.0366**			
*n* (response assessment population)	13	9	75	33
Best overall response—*n* (%)				
Complete response	0	0	1 (1)	0
Partial response	0	0	15 (20)	0
Stable disease	3 (23)	1 (11)	17 (23)	11 (33)
Progressive disease	6 (46)	4 (44)	37 (49)	21 (64)
Not evaluable	4 (31)	4 (44)	5 (7)	1 (3)
Objective response rate—*n* (%)	0	0	16 (21)	0
Disease control rate—*n* (%)	3 (23)	1 (11)	33 (44)	11 (33)

*CI* confidence interval, *Na* serum sodium level, *OS* overall survival, *P-meta* peritoneal metastasis, *PFS* progression-free survival, *RMST* restricted mean survival time

^a^Statistically significant differences (*p* < 0.05) are highlighted in bold. The definition of ∆∆RMST is described in “Methods”

## Discussion

The ATTRACTION-2 study demonstrates that nivolumab monotherapy provides benefit to advanced G/GEJ cancer patients who previously received at least two chemotherapy regimens [13]. In BaPoFi analysis, we found that combinations of the serum sodium level and either the WBC or NLR effectively classified patients into subgroups associated with low and high benefit from nivolumab. In addition, further exploratory analyses showed that the combination of age, peritoneal metastasis, and serum sodium level defined the markedly low- and high-benefit groups with the largest ∆∆RMST, suggesting that this triple-factor combination would be the most effective predictor of markedly low and high benefit from nivolumab among any combinations tested in this study. These classifications may be useful because these data are available to clinicians in daily practice.

A combination of the serum sodium level (lower vs. normal range) and WBC (higher vs. normal range) and that of the serum sodium level and NLR (higher tertile vs. middle/lower tertile) were associated with significant differences in the ∆∆RMST of both OS and PFS between the low- and high-benefit groups. Low serum sodium levels can occur from various disorders, including dehydration, diarrhea, hypothyroidism, concentrated urine, and heart and kidney failures [20], representing generally worse conditions. Low serum sodium levels have also been reported as a negative prognosis indicator associated with poor PFS for various cancer types [21–23]. In addition, in vitro analyses and in vivo analyses using mouse models have shown that increased serum sodium levels induce interleukin-17-producing helper T lymphocytes and impair functions of regulatory T lymphocytes [24–26], paradoxically suggesting that a low serum sodium level reduces immune capacity. Indeed, a low baseline concentration of sodium in the serum was associated with poor outcomes of ICI therapy for non-small cell lung carcinoma patients [27]. On the other hand, a higher WBC counts and higher NLR would generally account for higher inflammatory states, and a lower lymphocyte level would represent lower immune competency. It has been reported that a higher NLR is an indicator for poor PFS and OS for several cancers, including G/GEJ cancer, treated with ICIs [28–31], and a lower NLR is associated with better response to ICIs [29, 31]. Therefore, it is conceivable that a combination of a low serum sodium level with either a high WBC or high NLR, which represents a low capability of immunosurveillance, would be associated with poor benefit from nivolumab.

Our exploratory analysis showed that the triple-factor combination of an age of 60 years, peritoneal metastasis, and serum sodium levels effectively classified patients into the markedly low- and high-benefit groups and others. Younger patients with gastric cancer at any stage generally had an equivalent or even better OS compared with elderly patients [32–34]. However, when limited to patients with stage IV gastric cancer, some studies showed that OS in younger patients was significantly shorter than that in elderly patients [34]. Some studies also suggested that disease-free survival after surgery of gastric cancer was significantly shorter in younger patients than in elderly patients [32]. Consistently, the RMST value of PFS in patients younger than 60 years was remarkably smaller than that in patients older than 60 years in this study. Considering most patients enrolled in ATTRACTION-2 had recurrent or stage IV gastric cancer, these observations suggest that advanced gastric cancer may be aggressively exacerbated in younger patients, particularly in a salvage treatment setting. Therefore, these patients could receive poor benefit from nivolumab since ICI therapy often induces delayed responses [18, 35]. This study also showed that peritoneal metastasis as a single classification factor was significantly associated with low benefit in OS, but not in PFS, from nivolumab. Consistently, peritoneal metastasis was shown to be associated with poor prognosis in patients with gastric cancer [36, 37], suggesting aggressive tumor progression in these patients. Taking these arguments together, tumors in younger patients with peritoneal metastasis under a generally worse condition including a low immune capacity may rapidly and aggressively progress, resulting in little benefit from nivolumab.

The objective response rate of 21% in the nivolumab arm of the markedly high benefit group classified by the combination of three factors was comparable to or even higher than those of 15% and 25% in patients with ≥ 1 and ≥ 10 PD-L1 combined positive score, respectively, who were treated with pembrolizumab monotherapy in the KEYNOTE-062 study [13, 38]. Considering different treatment lines evaluated in ATTRACTION-2 (third-line or later) and KEYNOTE-062 (first-line), the criteria selecting the markedly high-benefit group may be promising. On the other hand, the disease control rate of 23% in the nivolumab arm of the markedly low-benefit group was comparable to that (25%) in the placebo arm of the whole study population [13], but was higher than that (11%) in the placebo arm of the markedly low-benefit group. Thus, small but certain benefit from nivolumab might be expected even in the markedly low-benefit group.

Recent researches have been paid attention to biomarkers to enrich the patients expected to have high efficacy of ICIs. However, in ATTRACTION-2, survival benefit of nivolumab was proved in all randomized patients. Therefore, it is not ethical to limit the indication of nivolumab to the enriched patients with high efficacy, excluding patients expected to have small benefits. In clinical practice in this situation, markers to predict patients with low benefit is clinically useful, especially for avoiding early progression.

We acknowledge several limitations in this study. First, this study retrospectively compared patient subgroups classified using multiple factors in an exploratory fashion, raising an issue of multiplicity. Internal validation could not be considered. Any statistically significant differences in this study may cause by chance because no adjustments for multiplicity were performed. In particular, the exploratory analyses assessing any double-factor combinations and the triple-factor combination could be far exploratory. Therefore, it is critical to confirm our findings in a future prospective clinical studies. Second, this study could not consider promising biomarkers including PD-L1 expression, tumor mutation burden, and CD8^+^ T lymphocytes because these data were available only in a limited number of patients in ATTRATION-2. Third, this study focused on nivolumab treatment as salvage therapy. Thus, potential predictors of benefit from nivolumab in earlier treatment lines may be different from those identified in this study. Fourth, we did not consider other cutoff values that would be more appropriate.

In conclusion, this exploratory analysis extracted several double-factor combinations such as the combination of serum sodium levels and either the WBC or NLR as potential predictors of benefit from nivolumab as salvage therapy in advanced G/GEJ cancer patients. In addition, patients younger than 60 years with peritoneal metastasis and with low serum sodium levels may expect markedly low benefit from nivolumab whereas elderly patients without peritoneal metastasis with normal serum sodium level may expect markedly high benefit from nivolumab. Future clinical studies with appropriate designs are warranted to confirm these hypotheses.

## Supplementary Information

Below is the link to the electronic supplementary material.

Supplementary file1 (PDF 1023 KB)
