# Supplementary material for: Exploration of predictors of benefit from nivolumab monotherapy for patients with pretreated advanced gastric and gastroesophageal junction cancer: post hoc subanalysis from the ATTRACTION-2 study
Source: Gastric Cancer. 2021 Sep 4;25(1):207–17. doi: 10.1007/s10120-021-01230-4 (PMC8732926; doi:10.1007/s10120-021-01230-4)
Supplement: Supplementary file 1 — Supplementary file1 (PDF 1023 KB) [file 10120_2021_1230_MOESM1_ESM.pdf]

Online Resource Tables and Figures

Gastric Cancer

Exploration of predictors of benefit from nivolumab monotherapy for patients with pretreated advanced gastric and gastroesophageal junction cancer: Post-hoc subanalysis from the ATTRACTION-2 study

Yoon-Koo Kang, Satoshi Morita, Taroh Satoh, Min-Hee Ryu, Yee Chao, Ken Kato, Hyun Cheol Chung, Jen-Shi Chen, Kei Muro, Won Ki Kang, Kun-Huei Yeh, Takaki Yoshikawa, Sang Cheul Oh, Li-Yuan Bai, Takao Tamura, Keun-Wook Lee, Yasuo Hamamoto, Jong Gwang Kim, Keisho Chin, Do-Youn Oh, Keiko Minashi, Jae Yong Cho, Masahiro Tsuda, Hiroki Sameshima, Li-Tzong Chen, and Narikazu Boku

Corresponding author: Yoon-Koo Kang, MD, PhD. Department of Oncology, Asan Medical Center, University of Ulsan College of Medicine, Seoul, South Korea.

Online Resource Table S1 Covariates and their cutoff values.

Covariates	Cutoff values
Age	[<50 vs. ≥50] or [<55 vs. ≥55] or [<60 vs. ≥60] or [<65 vs. ≥65] or [<70 vs. ≥70]
Sex	Male vs. Female
Body mass index	[<17.0 vs. ≥17.0] or [<18.5 vs. ≥18.5]
ECOG PS	0 vs. 1
History of alcohol consumption	Never vs. Former vs. Current
History of smoking	Never vs. Former vs. Current
Lesion site	Gastric vs. gastroesophageal junction
Borrman's classification	Type 4 vs. Others
Lauren classification	Diffuse vs. Others
Metastatic organ[a]	Yes vs. No
Number of metastatic organs	[<2 vs. ≥2] or [<3 vs. ≥3] or [<4 vs. ≥4]
Number of previous regimen	[≤2 vs. >2] or [≤3 vs. >3] or [≤4 vs. >4]
Gastrectomy	Yes vs. No
Radiotherapy	Yes vs. No
Prior therapy[b]	Yes vs. No
Last therapy[b]	Yes vs. No
Best response to the last regimen	Effective vs. Non-effective
Hemoglobin	Lower than institutional standard value vs. Normal
White blood cells	Upper than institutional standard value vs. Normal
Neutrophils	Median or Tertile[c]
Lymphocytes	Lower than institutional standard value vs. Normal
Eosinophils	Median or Tertile[c]
Basophils	Median or Tertile[c]
Monocytes	Median or Tertile[c]
Platelets	Upper than institutional standard value vs. Normal
Albumin	Lower than institutional standard value vs. Normal
Alkaline phosphatase	Upper than institutional standard value vs. Normal
Total bilirubin	Upper than institutional standard value vs. Normal
Protein	Lower than institutional standard value vs. Normal
Creatinine	Upper than institutional standard value vs. Normal
Lactate dehydrogenase	Upper than institutional standard value vs. Normal
Blood urea nitrogen	Upper than institutional standard value vs. Normal
Sodium	Lower than institutional standard value vs. Normal
Potassium	Lower than institutional standard value vs. Normal
Chloride	Lower than institutional standard value vs. Normal
C-reactive protein	<1 mg/dL vs. ≥1 mg/dL
Neutrophil-lymphocyte ratio	Median or Tertile[c]
Platelet-lymphocyte ratio	Median or Tertile[c]
C-reactive protein-albumin ratio	Median or Tertile[c]
Prognostic nutritional index	Median or Tertile[c]

ECOG PS, Eastern Cooperative Oncology Group Performance Status.

[a]Metastases to the bone, brain, liver, lung, lymph node, peritoneum, preural tissue, remnant stomach, and other organs were individually evaluated.

[b]Prior experiences with cisplatin, irinotevan, oxaliplatin, ramucirumab, taxanes, and trastuzumab were individually evaluated.

[c]Due to limitations of the conventional univariable analyses, the median was used as the cutoff value for these factors, whereas tertiles were used as the cutoff values of the factors in the BaPoFi analysis.

Online Resource Table S2 The factors showing significant associations with disease progression within 8 weeks in the nivolumab arm that were extracted by conventional logistic regression analysis.

**Factors [Cutoff]**	**N**	**Odds ratio**	**95% CI**
**Univariable logistic regression analysis**			
Age [<60 vs. ≥60]	330	2.03	1.28–3.21
Age [<65 vs. ≥65]	330	2.19	1.40–3.43
Age [<70 vs. ≥70]	330	2.55	1.51–4.30
History of alcohol consumption [Current vs. Former or Never]	330	0.39	0.20–0.79
Metastasis (Liver) [Yes vs. No]	330	1.66	1.06–2.59
Metastasis (Lung) [Yes vs. No]	330	0.54	0.31–0.95
Metastasis (Lymph node) [Yes vs. No]	330	0.55	0.34–0.86
Metastasis (Peritoneum) [Yes vs. No]	330	2.03	1.30–3.17
Number of metastatic organs [<2 vs. ≥2]	330	0.56	0.34–0.93
Last therapy (Ramucirumab) [Yes vs. No]	330	0.39	0.17–0.89
White blood cells [Upper vs. Normal]	330	1.98	1.06–3.70
Neutrophils [<Median vs. ≥Median]	330	0.60	0.38–0.93
Platelets [Upper vs. Normal]	330	1.89	1.04–3.44
Lactate dehydrogenase [Upper vs. Normal]	330	1.65	1.03–2.63
Sodium [Lower vs. Normal]	330	2.33	1.24–4.38
Chloride [Lower vs. Normal]	330	2.31	1.13–4.75
C-reactive protein [<1 mg/dL vs. ≥1 mg/dL]	329	0.53	0.34–0.83
C-reactive protein-albumin ratio [<Median vs. ≥Median]	329	0.48	0.31–0.76
Prognostic nutrition index [<Median vs. ≥Median]	330	1.69	1.08–2.64
**Multivariable logistic regression analysis**			
Age [<60 vs. ≥60]	330	2.16	1.15–4.05
Metastasis (Liver) [Yes vs. No]	330	2.09	1.11–3.93
Metastasis (Peritoneum) [Yes vs. No]	330	2.27	1.22–4.21

CI, confidence interval.

Online Resource Table S3 The factors showing significant associations with disease progression in the nivolumab arm that were extracted by conventional Cox analysis.

Factors [Cutoff]	N	Hazard ratio	*p* value
**Univariable Cox regression analysis**			
Age [<50 vs. ≥50]	330	1.50	0.0193
Age [<60 vs. ≥60]	330	1.59	0.0002
Age [<65 vs. ≥65]	330	1.37	0.0104
Metastasis (Liver) [Yes vs. No]	330	1.35	0.0147
Metastasis (Lymph node) [Yes vs. No]	330	0.66	0.0011
Metastasis (Peritoneum) [Yes vs. No]	330	1.52	0.0009
Number of metastatic organs [<2 vs. ≥2]	330	0.77	0.0497
Prior therapy (Ramucirumab) [Yes vs. No]	330	0.65	0.0338
Last therapy (Ramucirumab] [Yes vs. No]	330	0.59	0.024
White blood cells [Upper vs. Normal]	330	1.81	0.0002
Neutrophils [<Median vs. ≥Median]	330	0.74	0.0156
Platelets [Upper vs. Normal]	330	1.38	0.037
Alkaline phosphatase [Upper vs. Normal]	330	1.29	0.0345
Lactate dehydrogenase [Upper vs. Normal]	330	1.70	<0.0001
Sodium [Lower vs. Normal]	330	1.65	0.0025
Chloride [Lower vs. Normal]	330	1.58	0.0162
C-reactive protein [<1 mg/dL vs. ≥1 mg/dL]	329	0.76	0.0221
Neutrophil-lymphocyte ratio [<Median vs. ≥Median]	330	0.68	0.0019
Platelet-lymphocyte ratio [<Median vs. ≥Median]	330	0.76	0.023
C-reactive protein-albumin ratio [<Median vs. ≥Median]	329	0.76	0.0212
Prognostic nutrition index [<Median vs. ≥Median]	330	1.40	0.0054
**Multivariable Cox regression analysis**			
Age [<60 vs. ≥60]	330	1.77	<0.0001
Metastasis (Lymph node) [Yes vs. No]	330	0.67	0.0063
Metastasis (Peritoneum] [Yes vs. No]	330	1.45	0.0115
White blood cells [Upper vs. Normal]	330	1.51	0.0326
Lactate dehydrogenase [Upper vs. Normal]	330	1.64	0.0007

Online Resource Table S4 RMST analysis of progression-free survival for patient subgroups classified by multiple cutoff values of age.

Cutoff[a]	Low-benefit group		High-benefit group		ΔΔRMST[d,e]
	N[b]	ΔRMST[c,e]	N[b]	ΔRMST[c,e]	
[<50 vs. ≥50]	51/33	0.9 (-0.99–2.70)	279/128	**2.5 (1.47–3.54)**	1.7 (-0.47–3.77)
[<60 vs. ≥60]	134/65	1.0 (-0.01–2.03)	184/90	**2.9 (1.49–4.26)**	**1.9 (0.14–3.59)**
[<65 vs. ≥65]	180/89	**1.9 (0.73–3.12)**	138/66	**2.3 (0.86–3.83)**	0.4 (-1.49–2.32)
[<70 vs. ≥70]	242/119	**2.3 (1.24–3.36)**	76/36	1.4 (-0.39–3.22)	-0.9 (-2.97–1.21)

RMST, restricted mean survival time.

[a]Cutoff values listed on the left are classification values for the low-benefit group.

[b]Numbers of patients in the nivolumab/placebo arms.

[c]ΔRMST is defined as the difference in RMST between the nivolumab and placebo arms. ΔRMST (months) with 95% confidence interval is shown.

[d]ΔΔRMST is defined as the difference in ΔRMST between the low- and high-benefit groups. ΔΔRMST (months) with 95% confidence interval is shown.

[e]Statistically significant differences ($p < 0.05$) are highlighted in bold.

Online Resource Table S5 Top five factors in terms of the utility function identified in the BaPoFi analysis.

Rank	Factors	Class	
		**High benefit group**	**Corresponding low benefit group**
		**Overall survival**	
1	Na + WBC	Na: Normal<br>WBC: Normal	Na: Lower than standard<br>WBC: Higher than standard
2	Na + NLR	Na: Normal<br>NLR: Middle/Low	Na: Lower than standard<br>NLR: Highest tertile
3	Na + Neutrophils	Na: Normal<br>Neutrophils: Middle/Low	Na: Lower than standard<br>Neutrophils: Highest tertile
4	Na + CL	Na: Normal<br>CL: Normal	Na: Lower than standard<br>CL: Lower than standard
5	CL + NLR	CL: Normal<br>NLR: Mid/Low	CL: Lower than standard<br>NLR: Highest tertile
		**Progression-free survival**	
1	CL + NLR	CL: Normal<br>NLR: Middle/Low	CL: Lower than standard<br>NLR: Highest tertile
2	Na + NLR	Na: Normal<br>NLR: Middle/Low	Na: Lower than standard<br>NLR: Highest tertile
3	Na + CL	Na: Normal<br>CL: Normal	Na: Lower than standard<br>CL: Lower than standard
4	Na + Platelets	Na: Normal<br>Platelets: Normal	Na: Lower than standard<br>Platelets: Higher than standard
5	Na + WBC	Na: Normal<br>WBC: Normal	Na: Lower than standard<br>WBC: Higher than standard

CL, serum chloride level; Na, serum sodium level; NLR, neutrophil-lymphocyte ratio; WBC, white blood cell count.

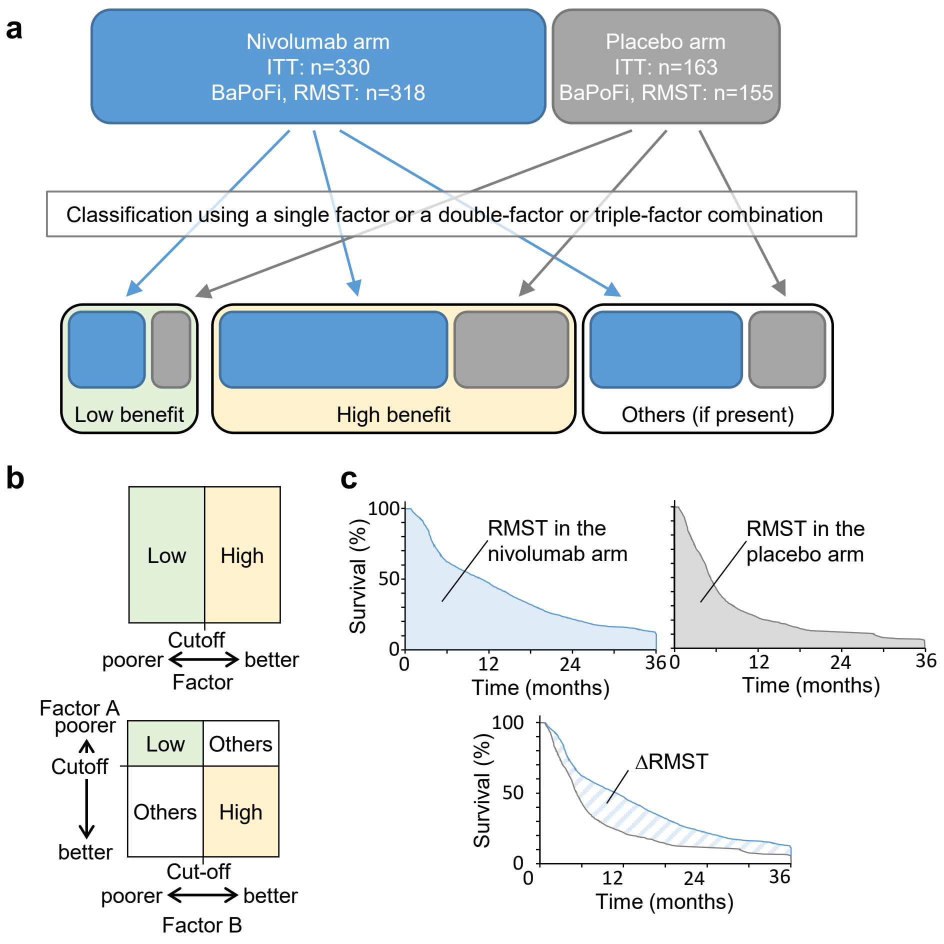

d

ΔΔRMST = ΔRMST (High-benefit group) - ΔRMST (Low-benefit group)

Online Resource Fig. S1

a Schematic representation of patient disposition. The number of patients in the intention-to-treat (ITT) population and that of patients who were included in the BaPoFi and restricted mean survival time (RMST) analyses are shown.

b (Upper panel) A single factor classified patients into two categories: a low-benefit group and a high-benefit group. (Lower panel) A double-factor combination classified patients into three categories: a low-benefit group, a high-benefit group, and the others.

c RMST is defined as the area under the curve. ΔRMST was defined as the difference of RMST between the nivolumab and placebo arms.

d ΔΔRMST was defined as the difference of ΔRMST between the low-benefit group and high-benefit group.

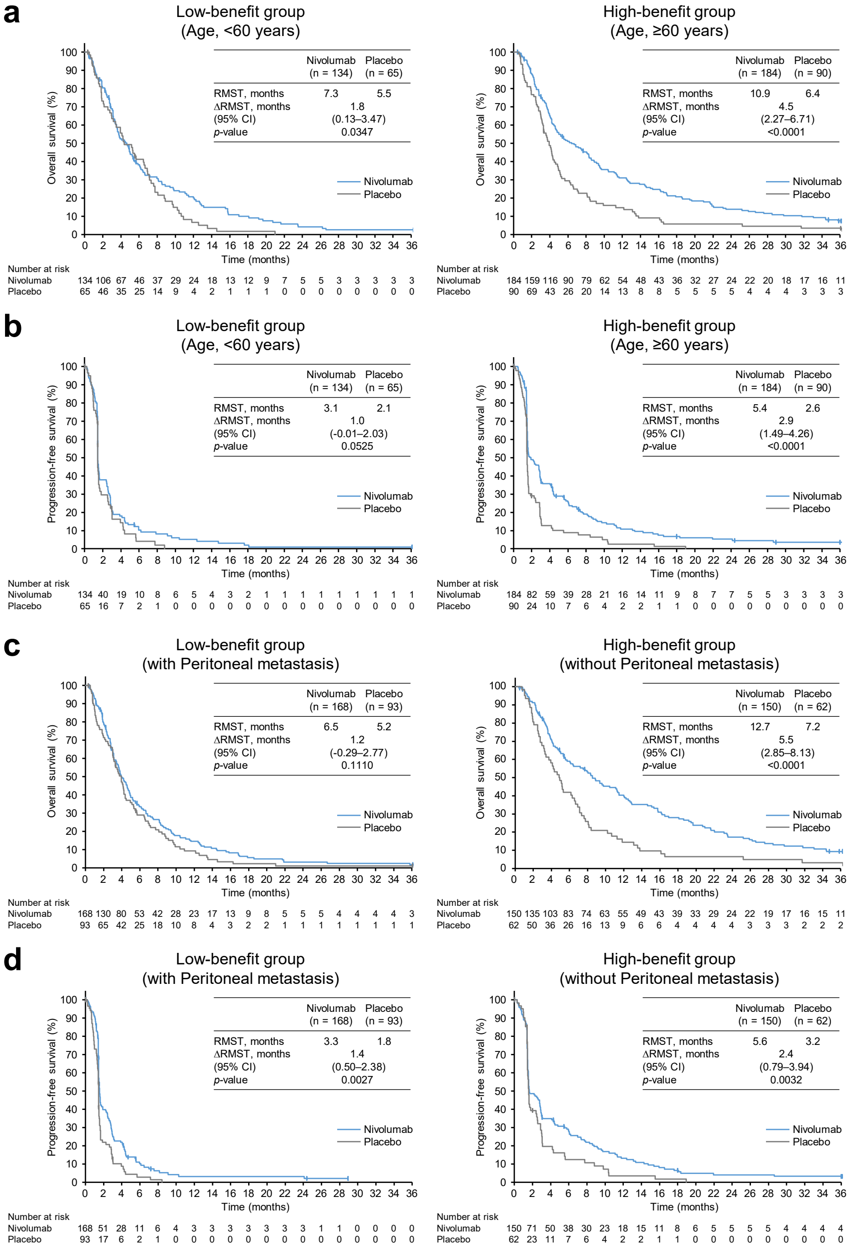

Online Resource Fig. S2 Classification by single factors of age (a, b) and peritoneal metastasis (c, d). The Kaplan–Meier curves of overall survival (a, c) and progression-free survival (b, d) with RMST values in low-benefit groups (left) and in high-benefit groups (right) are shown. CI, confidence interval; RMST, restricted mean survival time; ΔRMST, difference of RMST between the nivolumab and placebo arms.

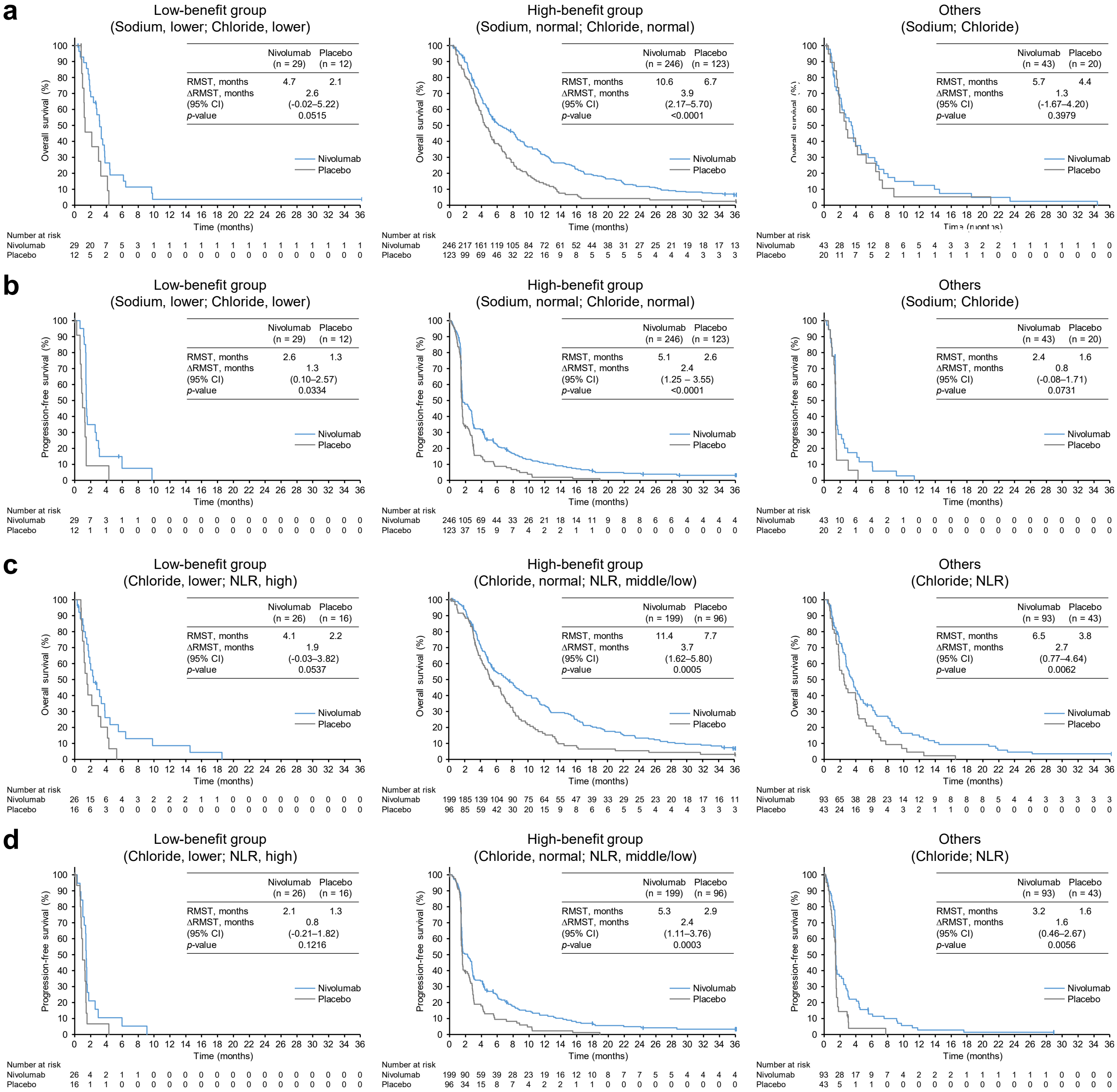

Online Resource Fig. S3 Classification by combinations of double factors of serum sodium level and serum chloride level (a, b) and of serum chloride level and neutrophil-lymphocyte ratio (NLR) (c, d). The Kaplan–Meier curves of overall survival (a, c) and progression-free survival (b, d) with RMST values in low-benefit groups (left), in high-benefit groups (right), and in others are shown. CI, confidence interval; RMST, restricted mean survival time; ΔRMST, difference of RMST between the nivolumab and placebo arms.
